# Frovatriptan 2.5 mg plus dexketoprofen (25 mg or 37.5 mg) in menstrually related migraine. Subanalysis from a double-blind, randomized trial

**DOI:** 10.1177/0333102414542290

**Published:** 2015-01

**Authors:** G Allais, G Bussone, V Tullo, P Cortelli, F Valguarnera, P Barbanti, G Sette, F D’Onofrio, M Curone, C Benedetto

**Affiliations:** 1 Women’s Headache Center, Department of Surgical Sciences, University of Turin, Italy; 2 Department of Clinical Neuroscience, National Neurological Institute Carlo Besta, Italy; 3 Neurological Clinic, Department of Biomedical and Neuromotor Sciences, University of Bologna, Italy and IRCCS Institute of Neurological Sciences of Bologna, Bologna, Italy; 4 Sestri Ponente Hospital “Padre Antero Micone”, Italy; 5 Unit for Treatment and Research of Headaches and Pain, IRCCS San Raffaele Pisana, Italy; 6 Sant’Andrea Hospital, Italy; 7 San Giuseppe Moscati Hospital, Italy

**Keywords:** Frovatriptan, dexketoprofen, migraine attacks, menstrually related migraine

## Abstract

**Purpose:**

The purpose of this article is to investigate the efficacy and safety of frovatriptan plus dexketoprofen 25 or 37.5 mg (FroDex25 or FroDex37.5, respectively) compared to that of frovatriptan 2.5 mg (Frova) in menstrually related migraine (MRM).

**Aim:**

The aim of this article is to analyze a subgroup of 76 women who treated an MRM attack in this multicenter, randomized, double-blind, parallel-group study.

**Methods:**

The primary end-point was the proportion of patients who were pain free (PF) at two hours. Secondary end-points included pain-relief (PR) at two hours and 48 hours sustained pain free (SPF).

**Results:**

PF rates at two hours were 29% under Frova, 48% under FroDex25 and 64% under FroDex37.5 (*p* < 0.05). PR at two hours was Frova 52%, FroDex25 81% and FroDex37.5 88%, while 48 hours SPF was 18% under Frova, 30% under FroDex25 and 44% under FroDex37.5.

**Conclusion:**

Combining frovatriptan+dexketoprofen produced higher PF rates at two hours compared to Frova while maintaining efficacy at 48 hours. Tolerability profiles were comparable.

## Introduction

The International Headache Society (IHS) defines menstrually related migraine (MRM) as attacks, in a menstruating woman, fulfilling the criteria for migraine without aura occurring on days –2 to +3 of menstruation in at least two of three menstrual cycles and additionally at other times of the cycle (1).

These migraine attacks represent a challenge both for the patient and the headache specialist as they have been shown to be more impairing and longer lasting than non-MRM attacks (2).

The first step of the acute treatment of MRM includes triptans followed by nonsteroidal anti-inflammatory drugs (NSAIDs) (3). If the response is less than optimal there is some evidence that a combination of triptan-NSAIDs is efficacious in menstrual migraine. The combination sumatriptan-naproxen showed to be better than placebo in two replicate randomized, controlled clinical trials of 621 adults with MRM and dysmenorrhea (4). There is also evidence that adding dexamethasone to rizatriptan in 35 women treating 190 MRM attacks is better than rizatriptan alone in improving efficacy although with a higher rate of adverse events (5). Combining a triptan and an NSAID has the potential to provide greater symptom relief than therapy with either drug alone because of the different pharmacodynamic targets of the two components: dilated blood vessels for triptans and inflammatory mediators such as prostaglandins (PGs) for NSAIDs (6,7). This could be particularly true in MRM, which is a difficult-to-treat type of migraine. Furthermore, PGs may play an important role in the pathogenesis of MRM (8,9).

Recently the combination of frovatriptan plus dexketoprofen (FroDex) at two different doses proved to be better than frovatriptan (Frova) alone in 314 patients with migraine with or without aura (10). The rationale for combining dexketoprofen with frovatriptan is linked to the intrinsic pharmacokinetic properties of the two drugs; dexketoprofen is rapidly absorbed and contributes to the early efficacy of the combination, whereas frovatriptan is absorbed more slowly but its effects persist for longer, so it provides sustained efficacy with fewer relapses (11).

The aim of this subgroup analysis was to compare the efficacy and tolerability of frovatriptan plus dexketoprofen in two different dosages with that of frovatriptan alone in women suffering from MRM.

## Methods

### Study population

Details regarding the study population have already been described in the main study (10).

Women with a history of MRM who treated a menstrual attack were selected for this analysis. MRM was defined, according to IHS criteria, as migraine without aura attacks in a menstruating woman, occurring on day 1 ± 2 (namely days −2 to +3) of menstruation in at least two out of three menstrual cycles and additionally at other times of the cycle (1).

### Study design

Details regarding the study design have already been described (10).

### Data analysis

This analysis was carried out in all normally menstruating women randomized to any of the three treatment sequences who had a positive history of MRM (with at least one MRM attack recorded in a diary in the preceding two months) and had treated one episode of menstrual migraine during the study.

The present subanalysis was predefined in the statistical analysis plan and original protocol of the main study (the patients’ diaries included a direct question for all normally menstruating women regarding whether they had treated the migraine attack with the study drug between two days before or three days after the day of beginning menstruation, which was taken as day 0).

The primary end-point was the proportion of patients pain free at two hours, defined as patients free of pain at two hours before any rescue medication (1). Secondary end-points were:

- rate of headache relief at two hours, defined as the percentage of patients with a decrease in headache from severe or moderate to mild or none within two hours (1);
- sustained pain-free rates, defined as the percentage of patients pain free within two hours with no use of rescue medication or recurrence within 48 hours (1);
- patients’ preference for treatments.

The primary variable was assessed by the Fisher Freeman Halton chi-square exact test using a 3 × 2 contingency table for tests of association and a 2 × 2 contingency table for comparisons between treatments.

The secondary end-points were assessed in the same way as the primary variable. The level of statistical significance was set at 0.05 for all analyses.

## Results

**Figure 1.**
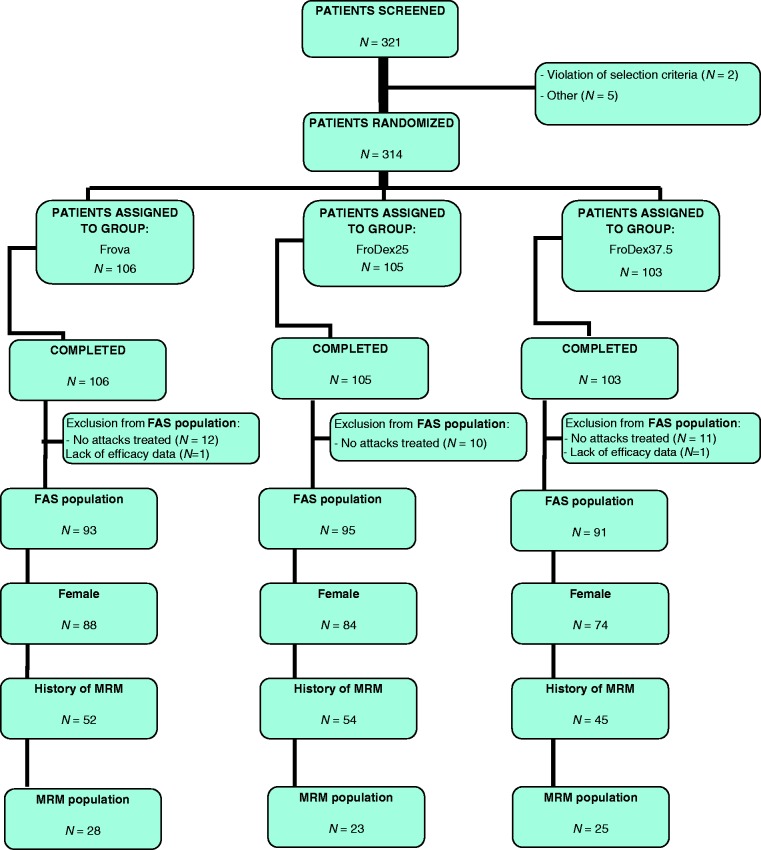
Flow diagram of participants throughout the study. FAS: Full analysis set.

A total of 248 women were enrolled in the main study, of whom 76 suffering from MRM were included in this analysis (Figure 1).

Table 1 shows baseline characteristics of the study populations. Migraine attacks in the MRM population had a higher mean Migraine Disability Assessment (MIDAS) score and higher proportions of patients with nausea, photophobia or phonophobia.

**Table 1.** Baseline characteristics.

	FAS (*n* = 279)	MRM (*n* = 76)	*p* value	Frovatriptan (*n* = 28)	FroDex 25 mg (*n* = 23)	FroDex 37.5 mg (*n* = 25)	*p*
	*n* (%)	*n* (%)	*FAS vs MRM*	*n* (%)	*n* (%)	*n* (%)	*Among treatment*
Ages (years)	38.8 ± 10	37.6 ± 7.9	= ***0.044***	37.8 ± 7.3	37.0 ± 9.7	37.8 ± 6.9	*0.931*
Height (cm, mean ± SD)	165 ± 7.1	163.8 ± 5.2	= ***0.016***	162.9 ± 4.3	164.0 ± 6.5	164.8 ± 4.8	*0.737*
Weight (kg, mean ± SD)	62 ± 10.4	57.9 ± 7.2	***<0.001***	57.1 ± 5.7	58.7 ± 8.4	58.0 ± 7.9	*0.409*
MIDAS score (mean ± SD)	23.9 ± 21.8	26.6 ± 23.2	= ***0.006***	24.0 ± 16.3	22.7 ± 12.8	25.4 ± 16.9	*0.843*
Intensity of attack							
Mild (*n*, %)	17 (6)	5 (6.6)		3 (10.7)	2 (8.7)	0	
Moderate (*n*, %)	177 (63)	42 (55.3)	*0.194*	15 (53.6)	14 (60.9)	13 (52.0)	*0.439*
Severe (*n*, %)	85 (31)	29 (38.2)		10 (35.7)	7 (30.4)	12 (48.0)	
Presence of nausea (*n*, %)	134 (48)	44 (57.9)	= ***0.051***	16 (57.1)	15 (65.2)	13 (52.0)	*0.648*
Presence of photophobia (*n*, %)	188 (67)	58 (76.3)	= ***0.054***	22 (78.6)	15 (65.2)	21 (84.0)	*0.292*
Presence of phonophobia (*n*, %)	173 (62)	59 (77.6)	**= *0.001***	19 (67.9)	16 (69.6)	24 (96.0)	= ***0.027***
Preventive therapy (*n*, %)							
Antidepressant	27 (10)	7 (9.2)	*0.970*	2 (7.1)	3 (13.0)	2 (8.0)	*0.240*
Antiepileptics	23 (8)	6 (7.9)	*0.897*	3 (10.7)	0	3 (12.0)	*0.744*
Beta-blocking agents	16 (5)	5 (6.7)	*0.586*	2 (7.1)	1 (4.3)	2 (8.0)	*0.868*
Triptan users (*n*, %)	71 (25.4)	15 (19.7)	*0.180*	6 (21.4)	5 (21.7)	4 (16.0)	*0.848*
NSAID users (*n*, %)	55 (19.7)	15 (19.7)	*0.995*	5 (17.9)	5 (21.7)	5 (20.0)	*0.941*

FAS: full analysis set; MRM: menstrually related migraine; FroDex: frovatriptan plus dexketoprofen; MIDAS: Migraine Disability Assessment; NSAID: nonsteroidal anti-inflammatory drug. = or < to the number to underline that values in bold are statistically significant.

### Overall efficacy of study drugs

#### Primary end-point

**Figure 2.**
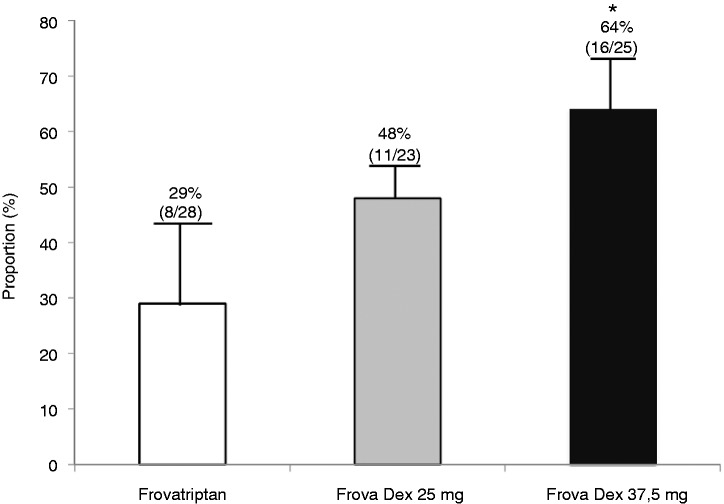
Percentage of patients pain free two hours after administration of frovatriptan (Frova), FroDex25 or FroDex37.5 in the 76 female with menstrually related migraine (MRM). Asterisks indicate a statistically significant difference (*p* < 0.05) between the group treated with Frova alone and the group treated with FroDex37.5. FroDex: frovatriptan plus dexketoprofen.

The overall comparison among treatments showed a statistically significant difference among the percentages of patients pain free at two hours in the different treatment groups (*p* < 0.05). The proportion of pain-free patients at two hours was 29% (nine of 28) under Frova, 48% (11/23) under FroDex25 and 64% (16/25) under FroDex37.5 (*p* < 0.05) (Figure 2).

#### Secondary end-points

The proportions of pain relief patients felt at two hours were 52% (13/28) for Frova, 81% (17/23) for FroDex 25 and 88% (22/25) for FroDex 37.5.

The proportions of patients who were sustained pain free at 48 hours were 18% (five of 28) under Frova, 30% (seven of 23) under FroDex25 and 44% under FroDex37.5 (11/25).

The treatment was judged excellent or good by 47% (13/28) in the Frova group, 61% (14/23) in the FroDex25 group and 80% (20/25) in the FroDex37.5 group.

### Tolerability

Overall, 14 treatment-emergent adverse events (three drug related) were reported in the MRM population: two with Frova (0 drug related), eight with FroDex25 (two drug related) and four with FroDex37.5 (one drug related). No events caused withdrawal from the study.

## Discussion

This subanalysis of a direct comparative pilot study showed that the combination of frovatriptan + dexketoprofen 25 mg or 37.5 mg was effective in the treatment of MRM, while maintaining a good tolerability profile.

Our most relevant finding was that the primary end-point, pain-free rate at two hours, was significantly higher with combination therapy than with Frova (29%) reaching 48% in the FroDex25 group and 64% in the FroDex 37.5 group. Interestingly the results obtained with the combination therapy are in line with those obtained with sumatriptan+naproxen sodium (pain free at two hours: 42%–52% with combination therapy vs 22%–23% with placebo) (4), although direct comparison studies of the two combination treatments are lacking.

As far the secondary end-points are concerned, the results of pain relief obtained at two hours were similar, showing a faster onset of action of the combination therapy. A sustained pain-free status is today considered the ideal migraine treatment response and the hardest end-point achievable in clinical studies. Sustained pain-free definition was calculated according to IHS guidelines; to our knowledge these are the first data published using this definition (1). The 48 hours’ sustained pain-free rates under combination therapy seem to confirm that it maintains a sustained effect over the 48 hours.

Interestingly, preference expressed by patients for the combination therapy showed a similar trend to the efficacy end-points results; we must underline that treatment was given in a double-blind fashion. All the study treatments were safe and well tolerated and the rate of treatment-related adverse events was low.

Notably, the baseline characteristics of the MRM population showed that these women had a higher MIDAS score and a higher proportion of associated symptoms than did the general female population of the main study, confirming that MRM attacks are more disabling and difficult to treat (2).

The results of this study should be interpreted in the context of its limitations. First, this is a subanalysis of a direct comparative pilot study and even if the statistical analysis plan and original protocol of the main study were predefined, this could be a limitation. Moreover, an a priori sample size was not calculated and post-hoc power was below 80%. This underpowered analysis was carried out with the clear purpose to create hypotheses that could be tested in a much larger trial.

A second limitation is that the tablets of frovatriptan, dexketoprofen and placebo were over-encapsulated separately. The third limitation is that our study did not include a dexketoprofen arm, but we must underline that the purpose of developing a combination therapy is to maximize both the pain-free rate at two hours and the sustained pain-free rate at 48 hours; dexketoprofen is well known to have a short half-life and its sustained effect is probably not relevant. Finally, our study did not include a placebo arm and for the secondary end-points lacked a correction for multiple statistical testing.

## Conclusions

In this sub-population analysis, we have shown that the association of FroDex may be better than Frova alone in the treatment of MRM and may be an additional option in this difficult-to-treat type of migraine.

In the near future we expect that more and more combinations of a triptan and an NSAID will be approved. The therapeutic synergism between these two antimigraine drug classes will help achieving a more prompt and consistent pain relief and will reduce the disability associated with the acute migraine attack. Taking into account the important role of PGs in the genesis of menstrual migraine, it is reasonable to assume that this type of migraine will particularly benefit from a combination of a triptan and an NSAID. Further large, ad-hoc clinical trials are needed confirm these results.

## Clinical implications

- A subgroup analysis on the combined use of frovatriptan plus dexketoprofen in menstrually related migraine was conducted.
- We sought to confirm the sound suggestion that combination therapy produces higher pain-free rates than frovatriptan monotherapy.
- Our results suggest that, in general terms, combination therapy has better results than monotherapy in most of the typical end-points for the treatment of migraine.
